# A randomized controlled trial of immediate implant placement comparing hydroxyapatite nano-coated and uncoated sandblasted/acid-etched implants using a digital surgical guide

**DOI:** 10.1186/s40729-024-00549-8

**Published:** 2024-06-05

**Authors:** Young-Chang Ko, Dongseob Lee, Ki-Tae Koo, Yang-Jo Seol, Yong-Moo Lee, Jungwon Lee

**Affiliations:** 1 Department of Periodontology and Dental Research Institute, School of Dentistry, Seoul National University, 101, Daehak-ro, Jongno-gu, Seoul, 03080 Republic of Korea; 2 National Dental Care Center for Persons with Special Needs, Seoul National University Dental Hospital, Seoul, Republic of Korea; 3 One-Stop Specialty Center, Seoul National University Dental Hospital, Seoul, Republic of Korea

**Keywords:** Digital surgical guide, Hydroxyapatite, Immediate implant placement, Implant surface modification

## Abstract

**Purpose:**

This study evaluated the implant stability, volumetric changes, and patient-reported outcome measures (PROMs) of hydroxyapatite (HA) nano-coated sandblasted/acid-etched (SLA) implants compared to uncoated SLA implants.

**Methods:**

Forty patients were recruited and randomly allocated to HA nano-coated SLA group (test, *n* = 20) and uncoated SLA group (control, *n* = 20) using single-blinded/block randomization. Implants were immediately placed in maxillary posterior region using a digital surgical guide. Insertion torque and implant stability quotient (ISQ) were measured at implant surgery and 1, 2, 3, and 4 months postoperatively. Intraoral scans, PROMs and soft tissue inflammation data were collected, and multivariable linear regression analysis of ISQ was performed.

**Results:**

In total, 48 implants (test; *n* = 24, control; *n* = 24) in 37 patients (test; *n* = 19, control; *n* = 18) were analyzed. Despite no significant between-group difference at surgery, the test group showed higher ISQ values than the control group at 2 (76.53 ± 4.17 vs. 71.32 ± 4.79, *p* < 0.01), 3 (77.45 ± 4.41 vs. 73.85 ± 4.69, *p* < 0.05), and 4 months (79.08 ± 2.96 vs. 73.43 ± 3.52, *p* < 0.0001) postoperatively. There were no significant differences in linear and volumetric changes, PROMs, and soft tissue inflammation analysis between two groups. The ISQ at implant surgery was influenced by age and diabetes mellitus (DM) at the implant level and DM and predicted total bone-to-implant contact area at the patient level.

**Conclusion:**

HA nano-coated SLA implants promoted favorable immediate implants stability during early osseointegration phase compared to uncoated SLA implants, but displayed similar dimensional changes, PROMs, and soft tissue inflammation outcomes.

**Trial registration:**

Clinical Research Information Service (CRIS), KCT0006364. Registered 21 July 2021, https://cris.nih.go.kr/cris/search/detailSearch.do?seq=24221&search_page=L.

**Supplementary Information:**

The online version contains supplementary material available at 10.1186/s40729-024-00549-8.

## Introduction

Over the past few decades, numerous studies have focused on the macrodesign of implants and surface modification to enhance the primary and/or secondary stability of dental implants [1, 2]. The most significant discovery was that the secondary stability of implants could be enhanced by altering the surface topography from a machined surface, leading to an increased success rate of dental implants, even in more challenging areas or conditions [3, 4]. Despite the clinical advancements achieved through surface modification, there remains a need to reduce the healing period before loading and to improve implant osseointegration at the bone graft site in challenging clinical situations, such as immediate implant placement with bone graft. Recently, with this perspective in mind, a variety of techniques have been developed to modify the implant surface to encourage cell adhesion, proliferation, and differentiation associated with osseointegration, and to boost the secretion of several cytokines to promote peri-implant osteogenesis [5].

Hydroxyapatite (HA) shares a similar composition with human bone, which has led to its long-standing use as a coating material for implant surfaces [6–8]. However, during long-term follow-up, a high rate of implant failure has been reported. This is attributed to the weak adhesive strength of HA to the implant surface and the detachment of the adhered HA layer, which is often due to the relative thickness of the HA layer [9–11]. With the advancement of technology, improvements have been made in adhesive strength and the ability to coat nano-level layers. Consequently, dental implants coated with biomaterials have become increasingly utilized.

A recent systematic review and meta-analysis that compared sandblasted/acid-etched (SLA) and SLA modified surface implants, found no significant difference between the two groups in terms of changes in implant stability following implant placement [12]. However, it is important to note that all the clinical trials included in the meta-analysis were conducted on healed ridges. In such a clinical context, the impact of implant surface modification could potentially be underestimated. Furthermore, SLA-modified implants may not be beneficial in clinical situations where there is ample bone quantity and quality, such as in a healed alveolar ridge. Therefore, it is suggested that clinical trials comparing SLA and SLA modified surface implants should be conducted in clinical situations where there is poor bone quality or insufficient bone quantity. This could include immediate implant placement in the maxillary posterior region, in order to determine the clinical significance of implants with SLA modification.

Meanwhile, surgical guides are essential for minimizing the impact of confounding factors such as the insertion angle, insertion direction, and 3-dimensional location of the implant, all of which can influence the long-term clinical outcome of the implant. In instances of immediate implant placement, positioning the implant correctly can be challenging. This difficulty often arises from the complexities of the drilling procedure following tooth extraction. Even with precise drilling, the implant tends to shift towards areas of poor bone quality or insufficient bone quantity during the final stages of implant installation. Given the high degree of difficulty associated with implant surgery, the use of a digital surgical guide becomes even more important.

The purpose of this study was to compare the clinical and radiological outcomes of HA nano-coated SLA implants with those of uncoated SLA-surface implants over a 10-year period. This was done in cases where the implants were immediately placed and bone grafting was concurrently performed in the maxillary posterior region. This paper presents the interim results at the 4-month mark after immediate implant placement, including a comparison of HA nano-coated SLA implants and uncoated SLA-surface implants in terms of implant stability, volumetric changes, and patient-reported outcome measures (PROMs). These factors will be examined in a 10-year prospective clinical trial.

## Methods

### Study design

**Fig. 1:**
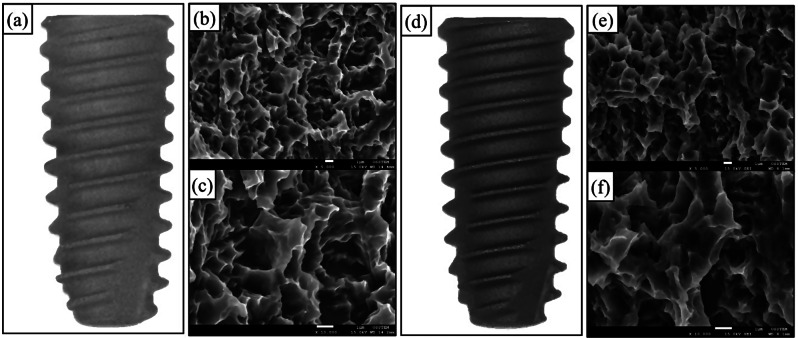
Macroscopic and scanning electron microscopy (SEM) images of control and test implants used in this study. (**a**-**c**) control implant, hydroxyapatite uncoated SLA (**d**-**f**) test implant, hydroxyapatite nano-coated SLA Magnification: X5,000 (**b**, **e**), X10,000 (**c**, **f**)

**Fig. 2:**
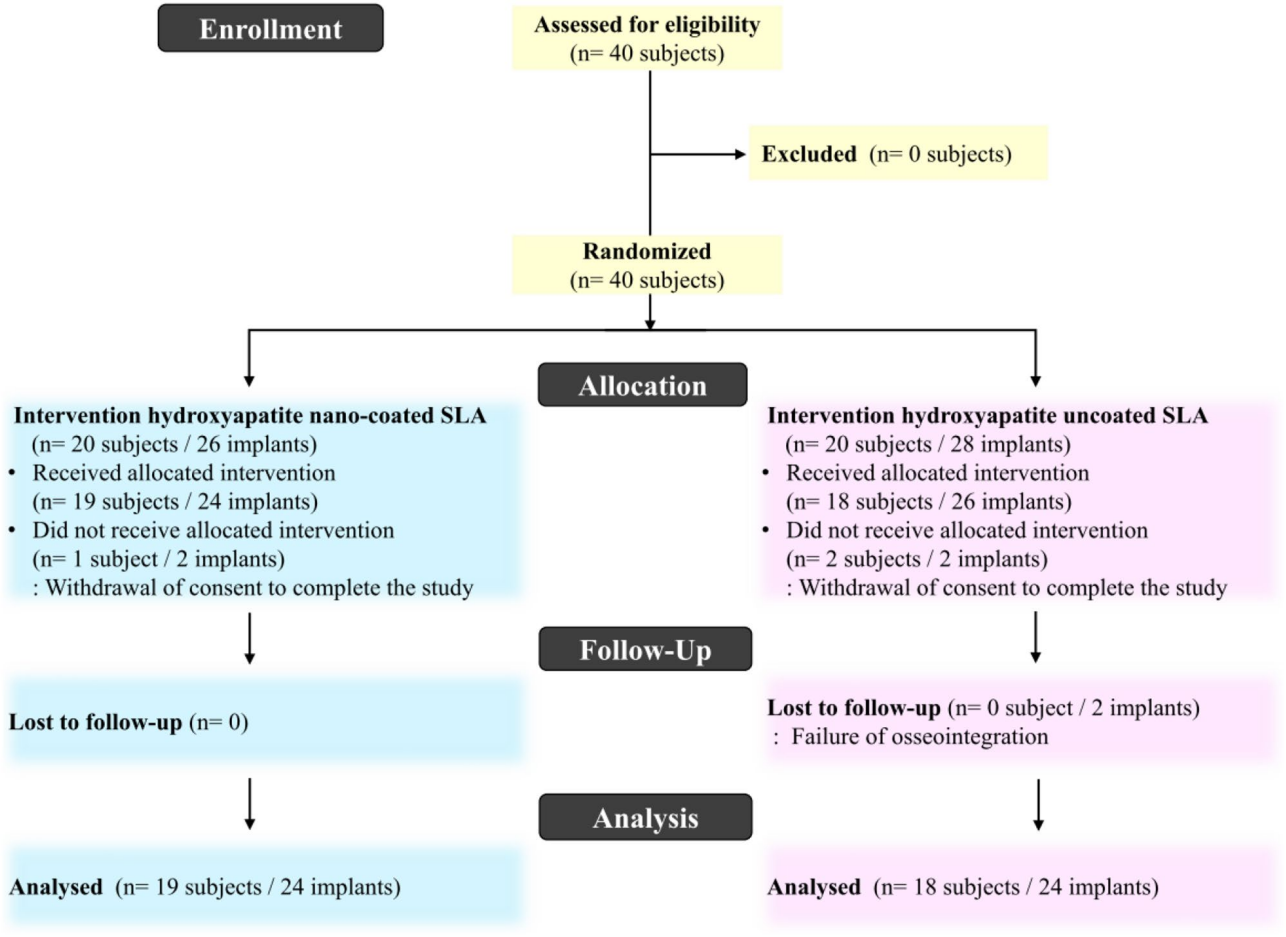
Consort flowchart

This randomized clinical trial was a single-blinded, two-group, parallel-group study. Participants were randomized on a 1:1 basis to either the HA nano-coated SLA group (test group, with a mean HA thickness of 10 nm and Ra of 2.5 μm; TSIII BA, Osstem Implant, Seoul, Korea) or the HA uncoated SLA group (control group, with Ra of 2.5 μm; TSIII SA, Osstem Implant, Seoul, Korea) (Fig. 1). The primary endpoint was evaluated up to four months after immediate implant placement. This prospective study received approval from the Institutional Review Board of the Seoul National University Dental Hospital (IRB No. CDE21007) and was registered with the Clinical Research Information Service (KCT0006364). All procedures were carried out in accordance with the Declaration of Helsinki, which outlines ethical principles for medical research involving human subjects [13]. Informed consent about the nature of the study was obtained from all participants prior to inclusion in the study. The manuscript was prepared according to the Consolidated Standards of Reporting Trials (CONSORT) guidelines [14]. The CONSORT flowchart of this study is presented in Fig. 2.

### Study setting and participants

Individuals were considered eligible for inclusion if they satisfied the following criteria: (1) they were 19 years of age or older; (2) they were scheduled to receive implants following tooth extraction in the maxillary posterior region; (3) they had a vertical bone height of 4 mm or more after tooth removal; (4) they exhibited no local pathology in the maxillary posterior region; (5) they voluntarily agreed to participate in the clinical trial and were willing to adhere to the study protocol.

Individuals were excluded from the study if they: (1) had an acute periodontal infection; (2) were anticipated to have difficulty achieving primary stability at the time of implant placement due to extensive bone loss (both buccal and palatal/lingual bone loss > 50%) resulting from periodontal disease, as determined by cone beam computed tomography (CBCT) evaluation; (3) were pregnant or lactating; (4) had uncontrolled hypertension or diabetes; (5) had a history of radiation therapy and/or chemotherapy in the head and neck area; (6) had serious cardiovascular, respiratory, kidney, liver, digestive, blood system, or neuropsychiatric diseases; (7) had a history of drug allergies; (8) had a severe depressive or anxiety disorder that could affect the clinical trial; (9) had engaged in drug or alcohol abuse within the past year; (10) had taken bisphosphonate drugs within the previous four months; (11) were smokers consuming more than one pack (20 cigarettes) per day [15, 16]; (12) had abnormal occlusion due to temporomandibular disorder; (13) were deemed unsuitable for participation in the study due to ethical considerations.

### Sample size calculation

The sample size was determined using the G * Power program version 3.1, developed by the University of Düsseldorf in Germany. Based on a previous study, it was projected that a difference of 3 between two groups in terms of implant stability would be clinically significant [17]. With a standard deviation (SD) of 3, an allocation ratio of 1:1, an alpha of 0.05, a power of 80%, and an anticipated exclusion rate of 15%, it was calculated that a total of 40 patients (20 patients per group) would be needed for the study.

### Recruitment, randomization, and blinding

Participants were recruited from the outpatient department of Seoul National University Dental Hospital. Study personnel conducted screenings of potential participants. The random number table method was employed, utilizing Excel’s RANDOM function to assign a random sequence. Once a subject was included after the screening, group assignments were made after verifying the encrypted file designated by an individual not involved in this study. Study subject identification codes were documented on the assignment table. To ensure balanced randomization between groups, the block size was set to 20, and randomization was conducted at a 1:1 ratio. Allocation concealment was achieved using sealed, opaque envelopes, which were filled by volunteers and opened on the day of the implant surgery following the implant osteotomy. The researcher responsible for data analysis was blinded to participant group assignments and outcome data. Subject allocation numbers and their corresponding data were anonymized, with the key held by one co-investigator (Y.M.L.) who was not involved in the data analysis.

### Preoperative preparation

**Fig. 3:**
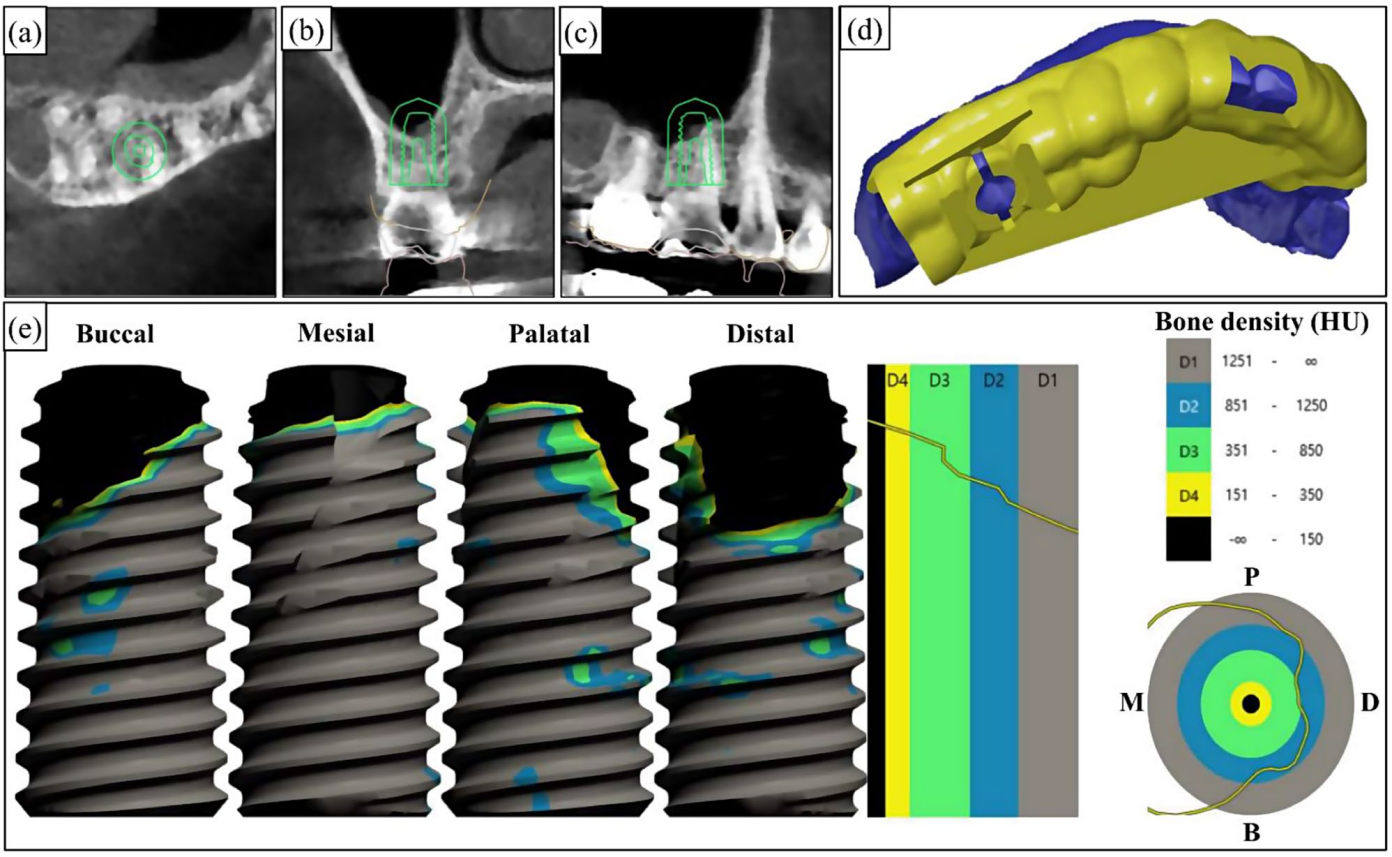
Flow of the digital surgical guide protocol. (**a**-**c**) Preoperative cone-beam computed tomography (CBCT) images and virtual implant position planning. (**d**) Virtual design and fabrication of the surgical guide. (**e**) Predicted bone-to-implant contact area according to bone density (HU; Hounsfield unit, D1; ≥1251 [gray], D2; 1250 − 851 [blue], D3; 351–850, [green] D4; 151–350, [yellow] None; ≤ 150 [black])

All subjects who met the inclusion criteria underwent preoperative cone-beam computed tomography (CBCT) (CS9300, Carestream Health, Rochester, NY, USA) for preoperative diagnosis. Digital Imaging and Communications in Medicine (DICOM) format files were then exported in preparation for implant surgery. A digital impression was captured using an intraoral scanner (Medit, Seoul, Korea), and Standard Tessellation Language (STL) files were subsequently exported. All implants were virtually planned using three-dimensional implant planning software (Implant Studio; 3Shape, Copenhagen, Denmark) by a surgeon (J.L.). The positioning of the dental implant was determined at the central axis of the virtual crown. The surgeon decided the length and diameter of the implants based on anatomical considerations and the intermaxillary relationship. In order to achieve sufficient primary stability, the position of implants was planned to include at least 3 mm of residual bone beyond the root apex, and implant platform level was predetermined below subcrestal 2 mm [18–20]. Implants with a 10 mm of length were planned, and in cases of inadequate residual bone height, implants were placed with transcrestal sinus augmentation. The diameters of implants were selected as 4 mm for premolar and 5 mm for molar teeth. Given the identical macrodesign of the test and control implants, the planning was performed without group allocation. Consequently, the surgeon was not aware of the subject’s allocation group. The predicted bone quality, defined as the expected bone contact area between the bone and implant, was recorded based on the Hounsfield unit (HU) values using the software (D1; ≥1251, D2; 1250 − 851, D3; 351–850, D4; 151–350, none; ≤ 150; Fig. 3). Following the surgical planning procedure, a surgical guide was fabricated using a 3D printer (Asiga UV Max, Sydney, Australia).

### Interventions

**Fig. 4:**
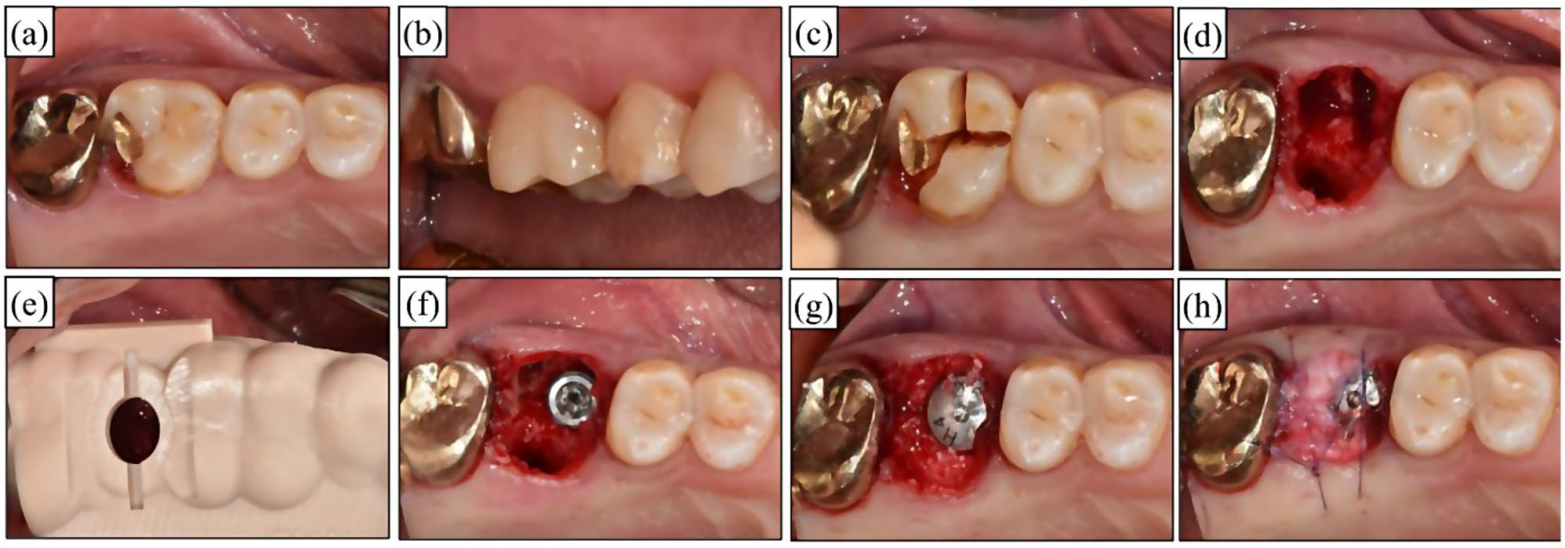
Overall surgical procedures. (**a**-**b**) Preoperative clinical photographs. (**c**) Tooth dissection for nontraumatic extraction. (**d**) Extraction socket after the debridement of granulation tissue. (**e**) Fitting digital surgical guide on surgical site. (**f**) Immediate implant placement. (**g**) Bone graft using deproteinized bovine bone mineral into marginal gap between socket wall and implant surface with/without transcrestal sinus augmentation. (**h**) Covering the surgical site with spongy type of collagen material and suturing

The surgical procedures are illustrated in Fig. 4 and Supplementary Fig. 1. Prior to surgery, patients were instructed to rinse their mouths with a 0.12% chlorhexidine solution (Bukwang Pharm, Seoul, Korea) for 1 min. The intraoral area was then scrubbed with povidone-iodine before the surgical procedure was conducted under local anesthesia using 2% lidocaine containing 1:100,000 epinephrine (Huons, Gyeonggi-do, Korea). Before tooth extraction, measurements were taken of tooth mobility, probing depth, gingival recession, the width of keratinized gingiva, and vestibule depth. Following tooth extraction, any granulation tissue in the extraction socket was curetted. Subsequently, a solution of doxycycline dissolved in saline was used for irrigation. A prefabricated surgical guide was applied without flap reflection, and the drilling procedure was performed, followed by implant placement. The marginal gap between the implant and the extraction socket wall was recorded. This marginal gap was filled with deproteinized bovine bone mineral (A-Oss, Osstem Implant, Seoul, Korea) and covered with a sponge-type collagen material (TERUPLUG, Olympus Terumo Biomaterials; Tokyo, Japan). At deficient residual bone height sites, transcrestal sinus augmentation was additionally performed with the gap filling procedure, and surgical site was secured with 5/0 Monosyn. All implant placement procedures were performed by an experienced periodontist (J.L.).

All participants were prescribed 100 mg of cefdinir and 650 mg of acetaminophen, to be taken every 8 h daily for a period of 7 days following surgery. Additionally, a 0.12% chlorhexidine solution (Bukwang Pharm, Seoul, Korea) was prescribed for use as a mouth rinse over the same 7-day period. Any use of medications other than those prescribed was duly recorded.

### Data collection

Data regarding the implant stability quotient (ISQ) were collected at the time of implant surgery, as well as 1, 2, 3, and 4 months post-implant placement. These were the primary time points for data collection. Additionally, to evaluate the correlation between the primary stability of the implant and the quality of the surrounding bone, the proportions of bone mineral density values were also measured using the HU values of the Misch bone-density classification [21] on buccal/palatal and mesial/distal surfaces of virtually planned implant fixtures (Fig. 3).

The plan was to conduct intraoral scans prior to surgery, as well as 1, 4, and 16 weeks post-implant surgery, at the time of prosthesis delivery, and 1, 3, 5, and 10 years after prosthesis delivery. This would allow for the analysis of volumetric changes at the implant sites. In this interim study, we analyzed data from before surgery and from 1, 4, and 16 weeks after implant surgery.

**Fig. 5:**
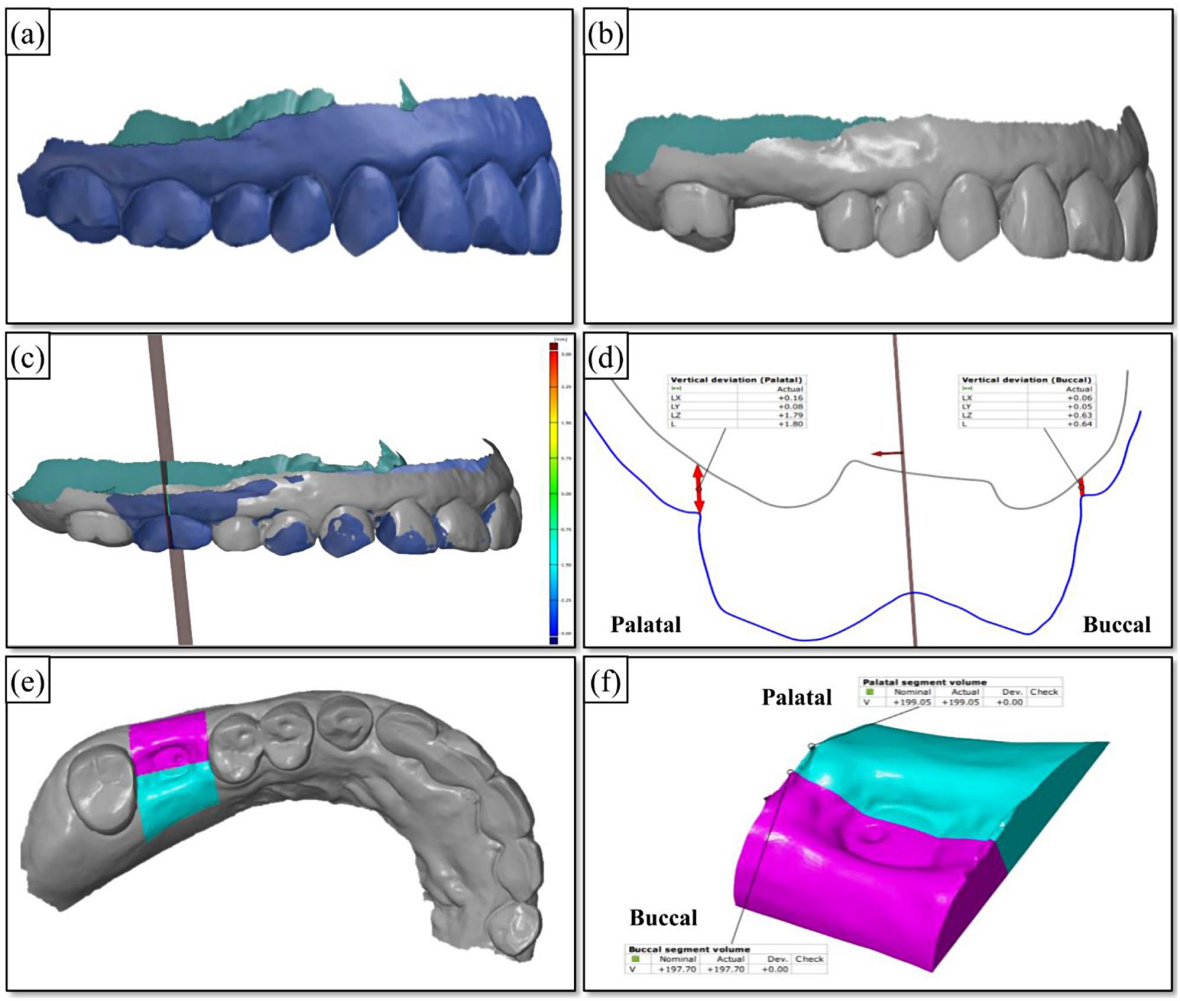
The outline of measurements of vertical deviations and volume changes before and after surgery. (**a**) Preoperative intraoral scan data. (**b**) Postoperative intraoral scan data. (**c**) Superimposition of pre-/post-operative intraoral scan data. The plane parallel to the long axis of the pre-existing tooth was set at the most-middle of buccal and palatal sides for vertical deviation measurement. (**d**) Cross-sectional image virtually dissected by the former plane. Vertical deviations (mm) were measured in a direction parallel to the former plane from buccal and palatal marginal gingiva. (**e**-**f**) Representative images of volume changes in buccal and palatal sides. Volumes were measured as absolute value (mm^3^) and converted into the relative percent (%) of post-/pre-operative volumes

Vertical linear alterations and three-dimensional volume changes at surgical sites were examined using intraoral scan data and a 3D geometric software program (GOM Inspect, GOM, Braunschweig, Germany), as per the methods outlined in previous studies [22, 23]. The two sets of intraoral scan data to be compared were automatically superimposed using the software program (Fig. 5). Changes in vertical linear distances between pre- and post-surgery (at 1, 4, and 16 weeks intervals) were measured in millimeters (mm) at the midpoint of the buccal and palatal marginal gingiva, parallel to the long axis of the pre-existing tooth. Concurrently, three-dimensional volume changes on the buccal and palatal sides were calculated as relative values (%). The horizontal boundary of the volume of interest (VOI) was determined between two contact points of teeth, and the vertical boundary was set to be 3 mm apically from the position of the central gingival margin. After the VOI was divided into buccal and palatal segments, referencing the extension line of the central pits, the volume of each segment was measured.

PROMs, including a subjective questionnaire and the Oral Health Impact Profile-14 (OHIP-14) [24], were obtained. The subjective questionnaire, which included questions about pain, swelling, discomfort with the implant placement procedure, satisfaction with the implant surgery, and willingness to undergo the same treatment in the event of future tooth loss, was administered on the day of surgery and again at 1 and 4 weeks post-surgery. Responses were recorded using visual analog scales (VAS, 0 = no pain, 100 = maximum pain). The OHIP-14 was administered 2 weeks prior to surgery, on the day of the operation, and 16 weeks post-surgery. Responses were recorded using a Likert scale (0 = never, 1 = hardly ever, 2 = occasionally, 3 = fairly often, 4 = very often).

The soft tissue inflammation score [25] was recorded at 1 and 4 weeks post-surgery using the following scale: 0 represents healthy peri-implant soft tissue; 1 indicates slight inflammation, characterized by a minor color change in the entire marginal or papillary soft tissue; 2 signifies mild inflammation, with no edema but a mild color change in the entire marginal and papillary soft tissue; 3 denotes moderate inflammation, marked by a glossy and reddened appearance, edema, and overgrowth of the marginal and papillary soft tissue; 4 corresponds to severe inflammation, characterized by significant redness, swelling, and overgrowth, spontaneous bleeding, or ulceration of the marginal and papillary soft tissue.

### Statistical analysis

The statistical analyses were conducted using SPSS software (version 25.0, Chicago, Illinois) and GraphPad Prism version 10.0.2 software for Windows (GraphPad Software, San Diego, CA, USA). Variables were expressed as either mean ± SD or median ± IQR, while categorical variables were represented by frequencies.

The changes in ISQ over time were evaluated using repeated-measures analysis of variance (rm-ANOVA), and Sidak multiple comparisons were conducted. A subgroup analysis of ISQ values (< 70 and ≥ 70 at the time of surgery) was performed using rm-ANOVA with Sidak multiple comparisons. The frequencies of ISQ and insertion torque values were compared between the control group and the test group using the chi-square test.

Changes in vertical deviation and volume at the buccal and palatal areas were evaluated using rm-ANOVA and Tukey multiple comparisons for within-group comparisons. The values between two groups at the same time point were compared using the unpaired t-test.

The subjective questionnaire, which included questions about pain and the sensation of swelling, was analyzed using rm-ANOVA with Tukey multiple comparisons test for intragroup comparisons. The unpaired t-test was performed for intergroup comparisons. The results of another subjective questionnaire, which included questions about discomfort with the implant placement procedure, satisfaction with implant surgery, and willingness to undergo the same treatment in the event of tooth loss, were compared between the two groups using the unpaired t-test. rm-ANOVA with the Tukey multiple-comparisons test was used to evaluate the significance of differences in each domain of the OHIP-14 at various time points, including 2 weeks before surgery, on the day of the implant surgery, and 16 weeks post-surgery. The soft tissue inflammation score was analyzed using the Mann-Whitney test for intragroup comparisons between two time points and intergroup comparisons at each timepoint.

A multiple linear regression model was utilized to determine whether candidate variables, including patient-, site-, and implant-related factors, were associated with the ISQ value at the time of implant surgery (V3).

## Results

### Demographic data

Detailed information about patient demographics and implant characteristics is presented in Table 1 and Supplementary Table 1. This study included a total of 37 patients (19 in the test group and 18 in the control group) and 50 implants (24 in the test group and 26 in the control group). These implants were immediately placed in the maxillary posterior extraction sockets. Out of the 26 implants in the control group, 2 implants were excluded due to failed osseointegration. Details about these failed implants can be found in Table 2. However, this did not affect the total number of patients in the study. The average age of patients in the test group was 55.84 ± 12.15 years, while in the control group it was 61.06 ± 9.19 years.

**Table 1:** Demographic data of the participants in this study

	Control	Test
**Patient-related factors**		
Number	18	19
Age (mean ± SD, years)	61.06 ± 9.19	55.84 ± 12.15
< 65	12	15
≥ 65	6	4
Sex		
Male	3	10
Female	15	9
Smoking		
No	17	16
Yes	1	3
Systemic diseases		
Hypertension	5	5
Diabetes mellitus	2	3
Hyperlipidemia	6	4
Hepatitis B virus	0	1
Osteoporosis	1	1
**Site-related factors**		
Tooth number	26	24
Location		
Premolar	10	11
Molar	16	13
Mobility		
Grade 0	6	9
Grade 1	7	3
Grade 2	2	2
Grade 3	11	10
Probing pocket depth (mm)	5.92 ± 2.09	5.78 ± 2.53
Recession depth (mm)	1.62 ± 1.32	1.28 ± 1.33
Clinical attachment level (mm)	7.55 ± 2.94	7.06 ± 3.15
Vestibular depth (mm)	7.65 ± 1.87	7.83 ± 1.86
Predicted total bone-to-implant contact area (PBA-T) (%)	56.24 ± 21.00	66.32 ± 27.84
Predicted D1/D2 bone-to-implant contact area (PBA-D1/D2) (%)	39.98 ± 21.66	56.45 ± 27.15
**Implant-related factors**		
Diameter		
4.0 mm	10	11
5.0 mm	16	13
Additional surgery		
Bone graft	21	18
Bone graft with transcrestal sinus augmentation	5	6
Design of prosthesis (number of patients)		
Single	10	14
2-abutment/2-unit prosthesis	4	1
2-abutment/3-unit prosthesis	4	3
3-abutment/4-unit prosthesis	0	1

**Table 2:** Description of failed implants

ID	Group	Age (years)	Sex	Smoking	Systemic disease	Tooth number	Mobility	PPD	REC	CAL	VD	PBA -T	PBA -D1/D2	Implant diameter	IT	ISQ	Timepoint of failure following implant surgery
LOC-18	Control	73	F	no	hyperlipidemia	14	3	12.00	4.17	16.17	9	70.62	57.36	4.0	30	45.75	2months
LOC-31	Control	63	F	no	HT	15	0	5.83	0.00	5.83	8	35.15	35.15	4.0	35	73.25	1month

ID, identification; PPD, probing pocket depth; REC, recession depth; CAL, clinical attachment level; VD, vestibular depth; PBA-T, predicted total bone-to-implant contact area; PBA-D1/D2, predicted D1/D2 bone-to-implant contact area; IT, insertion torque; ISQ, implant stability quotient

All intervention sites were located in the maxillary premolar (test; *n* = 11, control; *n* = 10) and molar (test; *n* = 13, control; *n* = 16) areas. The predicted marginal gap measurements were 4.46 ± 2.70 mm, 9.04 ± 3.88 mm, and 8.54 ± 4.24 mm (width, length, and depth, respectively) in the test group, and 4.23 ± 2.67 mm, 8.73 ± 4.64 mm, and 8.58 ± 3.72 mm in the control group. Furthermore, the predicted total bone-to-implant contact area (PBA-T) was 66.32 ± 27.84% in the test group and 56.24 ± 21.00% in the control group. The predicted D1/D2 bone-to-implant contact area (PBA-D1/D2) was 56.45 ± 27.15% in the test group and 39.98 ± 21.66% in the control group.

Implants with diameters of 4.0–5.0 mm were placed, and all marginal gaps between the extraction socket and the implant were filled with bone graft material. Additionally, transcrestal sinus augmentation surgery was performed on six implants in the test group and five implants in the control group.

### Description of failed implants

Two implants from two patients in the control group had to be removed 1 and 2 months after placement, respectively, due to failed osseointegration (Table 2). Both of these implants were part of multiple implant procedures. One of these implants was successfully replaced 4 months after the initial removal, resulting in successful osseointegration. The other implant was replaced 3 months after removal, but unfortunately, it failed again. However, 6 months after the removal of this second failed implant, a third attempt at implant replacement was successfully carried out.

### Primary outcome

#### Insertion torque and primary stability at implant surgery

The mean insertion torque in the test group was 32.92 ± 7.79 Ncm, compared to 34.81 ± 7.28 Ncm in the control group. The mean ISQ values at the time of implant surgery were 69.91 ± 7.21 in the test group and 71.26 ± 8.81 in the control group. No significant differences in insertion torque and primary stability were observed between the two groups (Table 3).

**Table 3:** Clinical data at implant surgery

	Control	Test	*p*-value
**Insertion torque**	26	24	0.6151
≤ 20 Ncm	2	4	
> 20 Ncm, ≤ 30 Ncm	8	7	
> 30 Ncm, ≤ 40 Ncm	15	13	
> 40 Ncm	1	0	
Mean ± SD	34.81 ± 7.28	32.92 ± 7.79	
**ISQ value**	26	24	0.4002
≤ 60 Ncm	2	2	
> 60 Ncm, ≤ 65 Ncm	1	4	
> 65 Ncm, ≤ 70 Ncm	5	5	
> 70 Ncm, ≤ 75 Ncm	7	8	
> 75 Ncm	11	5	
Mean ± SD	71.26 ± 8.81	69.91 ± 7.21	

ISQ, implant stability quotient

#### Implant stability

**Fig. 6:**
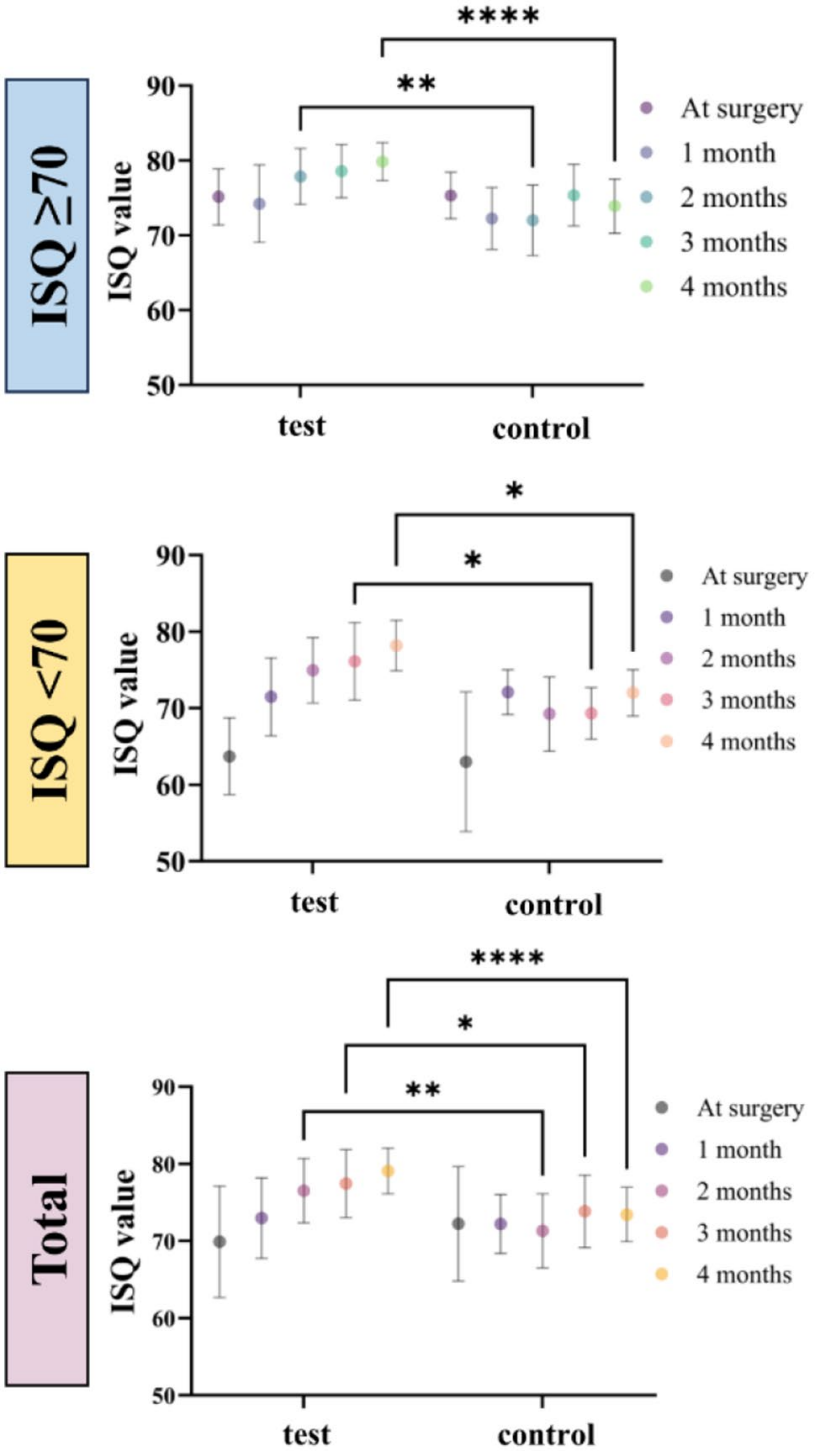
Implant stability between the test and control groups. Implant stability quotient (ISQ) values were measured at implant surgery day and 1, 2, 3, and 4 months after surgery. Data are presented as the mean ± SD. *p < 0.05, **p < 0.01, ****p < 0.0001

Generally, the test group demonstrated a trend of increasing ISQ over time, while the control group exhibited a curve where stability initially decreased before increasing again (Fig. 6). Among the implants with an initial ISQ value of 70 or higher, statistically significant differences were observed between the two groups at 2 months (77.87 ± 3.74 in the test group, 72.01 ± 4.71 in the control group, *p* < 0.01) and 4 months post-implant surgery (79.85 ± 2.53 in the test group, 73.90 ± 3.63 in the control group, *p* < 0.0001). For implants with an initial ISQ value below 70, significant differences were noted at 3 months (76.11 ± 5.09 in the test group, 69.33 ± 3.33 in the control group, *p* < 0.05) and 4 months after surgery (78.18 ± 3.29 in the test group, 72.00 ± 3.03 in the control group, *p* < 0.05). Overall, HA nano-coated SLA implants showed significantly improved implant stability at 2, 3, and 4 months post-surgery compared to uncoated SLA implants (76.53 ± 4.17 vs. 71.32 ± 4.79, *p* < 0.01 at 2 months; 77.45 ± 4.41 vs. 73.85 ± 4.69, *p* < 0.05 at 3 months; and 79.08 ± 2.96 vs. 73.43 ± 3.52, *p* < 0.0001 at 4 months, for the test group vs. the control group, respectively).

### Secondary outcomes

#### Vertical deviations

**Fig. 7:**
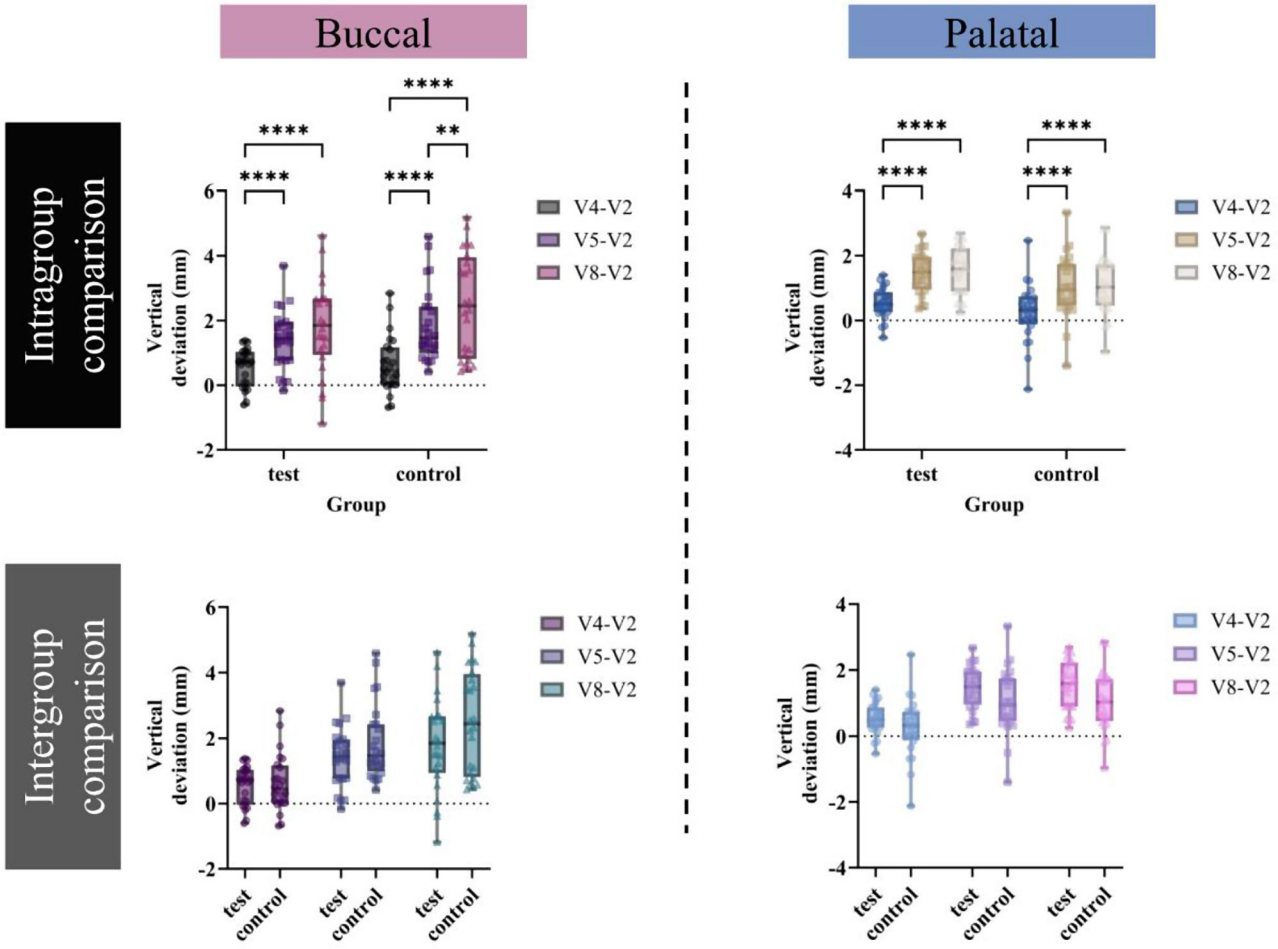
Intragroup and intergroup comparisons for vertical deviations on the buccal and palatal sides. Vertical deviations (mm) comparisons were conducted at 1 (V4), 4 (V5), 16 (V8) weeks after surgery. Positive value: to apical, negative value: to coronal. Data are presented as the mean ± SD. **p < 0.01, ****p < 0.0001

In the results of vertical deviations at 1 (V4), 4 (V5), and 16 (V8) weeks from the pre-surgery baseline (V2) (Fig. 7), two implants in the control group were excluded from this analysis due to failed osseointegration. In both groups, significant reductions in vertical bone height were observed on both the buccal and palatal sides, even after the bone graft procedure. These reductions increased over time. Notably, dramatic linear changes from the pre-surgery baseline were particularly evident at 4 and 16 weeks post-implant surgery. On the buccal side, vertical deviations from the pre-surgery baseline were 0.56 ± 0.60 mm, 1.42 ± 0.92 mm, and 1.81 ± 1.43 mm in the test group, and 0.63 ± 0.88 mm, 1.86 ± 1.16 mm, and 2.50 ± 1.59 mm in the control group (at 1, 4, and 16 weeks post-surgery, respectively). On the palatal side, vertical deviations were 0.54 ± 0.48 mm, 1.47 ± 0.64 mm, and 1.54 ± 0.74 mm in the test group, and 0.24 ± 0.90 mm, 1.04 ± 0.99 mm, and 1.07 ± 0.91 mm in the control group (at 1, 4, and 16 weeks post-surgery, respectively). However, in comparisons between the two groups, only minor differences were observed on both the buccal and palatal sides at all time points.

#### Three-dimensional volume changes

**Fig. 8:**
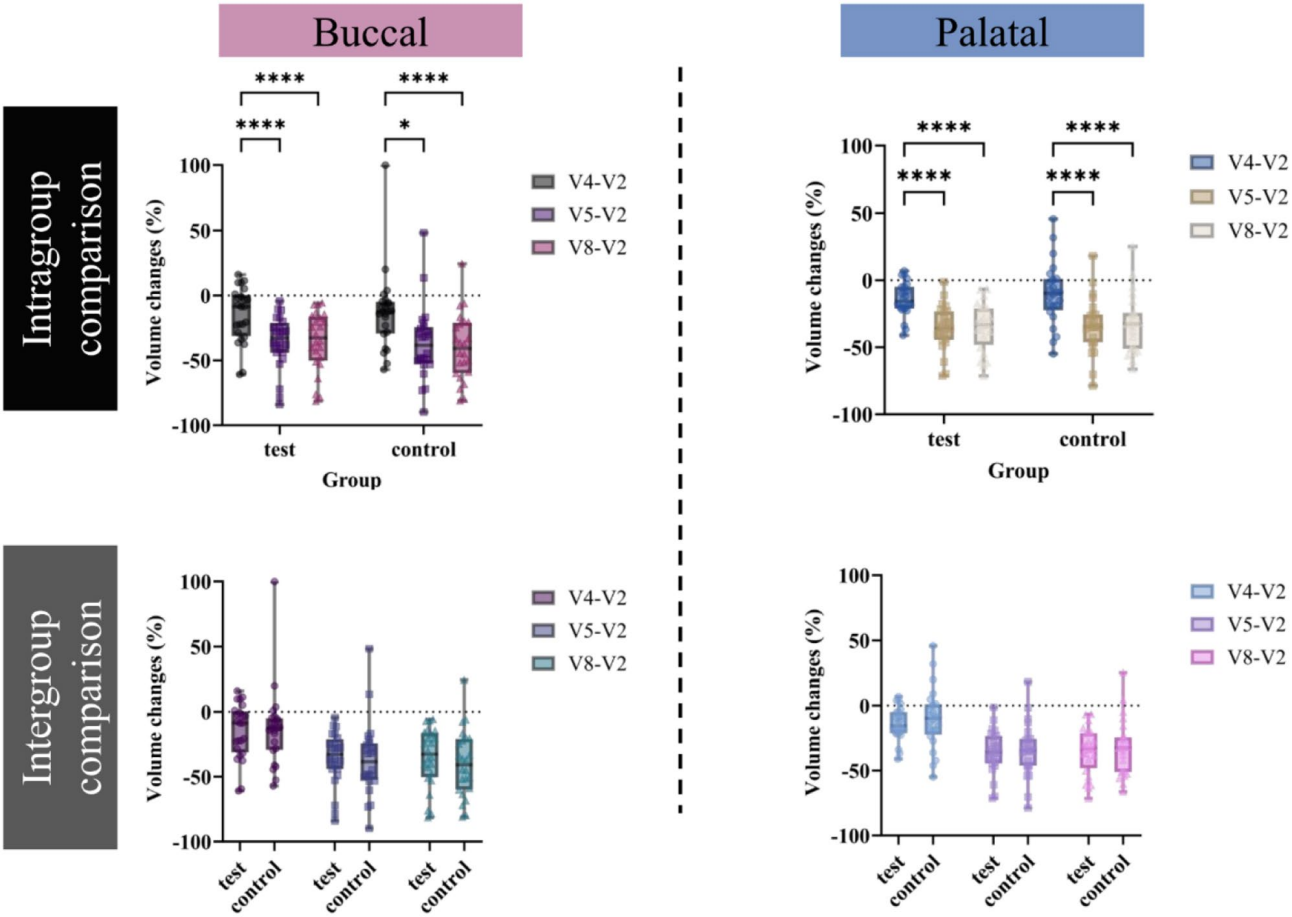
Intragroup and intergroup comparisons for volume changes on the buccal and palatal sides. Volume change (%) comparisons were conducted at 1 (V4), 4 (V5), and 16 (V8) weeks after surgery. Positive value: increase, negative value: decrease. Data are presented as the mean ± SD. *p < 0.05, ****p < 0.0001

In the results of volume changes at 1 (V4), 4 (V5), 16 (V8) weeks post-surgery, compared to pre-surgery measurements (V2) (Fig. 8), the ridge volume of the surgical sockets significantly decreased at 4 weeks post-surgery in both groups, with the volume reduction maintaining a similar level up to the 16-week mark. On the buccal side, the relative volume changes in the test group were 14.88 ± 21.26%, 35.67 ± 20.60%, and 35.29 ± 22.95% at 1, 4, and 16 weeks post-surgery, respectively. In the control group, the changes were 13.07 ± 30.49%, 40.36 ± 21.90%, and 40.07 ± 25.66% at the same time points. On the palatal side, the volume changes in the test group were 14.27 ± 12.68%, 35.71 ± 16.87%, and 34.93 ± 18.01% at 1, 4, and 16 weeks post-surgery, respectively. In the control group, the changes were 10.14 ± 23.27%, 33.95 ± 20.77%, and 33.07 ± 21.77% at the same time points. However, no statistically significant differences were observed between the groups at any of the time points.

#### Patient-reported outcome measures (PROMs)

**Fig. 9:**
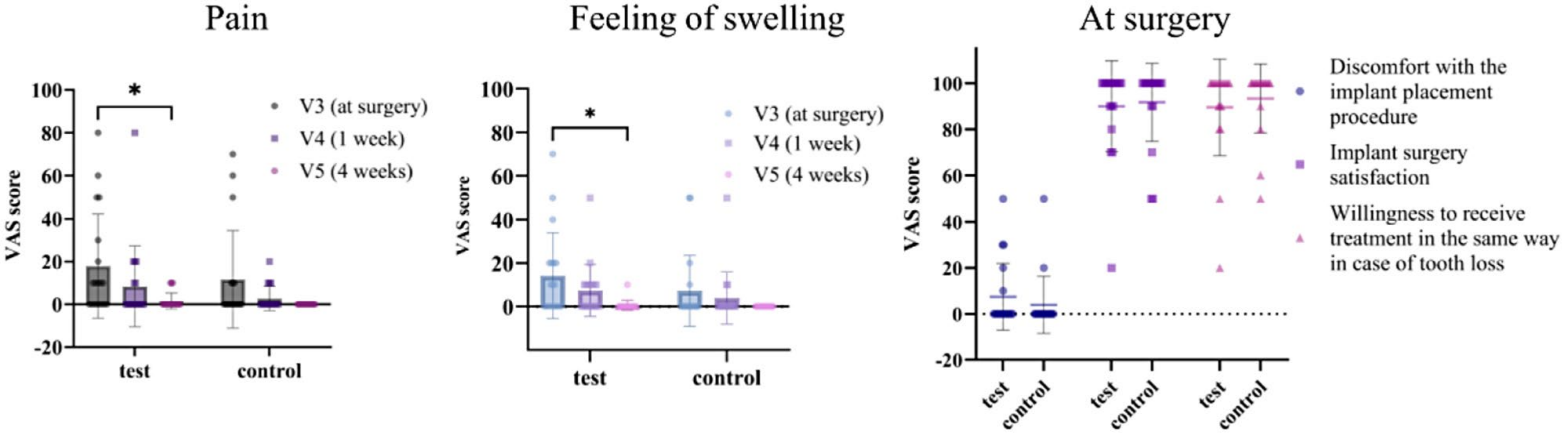
Visual analog scales (VAS) scores for pain, feeling of swelling at surgery (V3) and 1 (V4) and 4 (V5) weeks post-surgery, and for discomfort with the implant placement procedure, implant surgery satisfaction, willingness to receive treatment in the same way in case of tooth loss after surgery. Data are presented as the mean ± SD. *p < 0.05

A total of 37 patients (18 in the control group and 19 in the test group) responded to the subjective questionnaire at the time of surgery (V3), as well as 1 week (V4) and 4 weeks (V5) post-surgery, as depicted in Fig. 9. Overall, postoperative symptoms such as pain and swelling gradually decreased over time, reaching near-zero levels 4 weeks after surgery. There was a statistically significant decrease in the VAS scores for pain (17.89 ± 24.40 vs. 1.58 ± 3.75, *p* < 0.05) and swelling (1.42 ± 1.98 vs. 0.05 ± 0.23, *p* < 0.05) between V3 (at surgery) and V5 (4 weeks post-surgery) in the test group. However, no statistically significant difference was observed in the control group across the three timepoints. There were also no statistically significant differences between the test and control groups at each timepoint. Factors such as discomfort during the implant placement procedure, satisfaction with the implant surgery, and willingness to undergo the same treatment in the event of tooth loss showed no differences in both within-group and between-group comparisons.

**Fig. 10:**
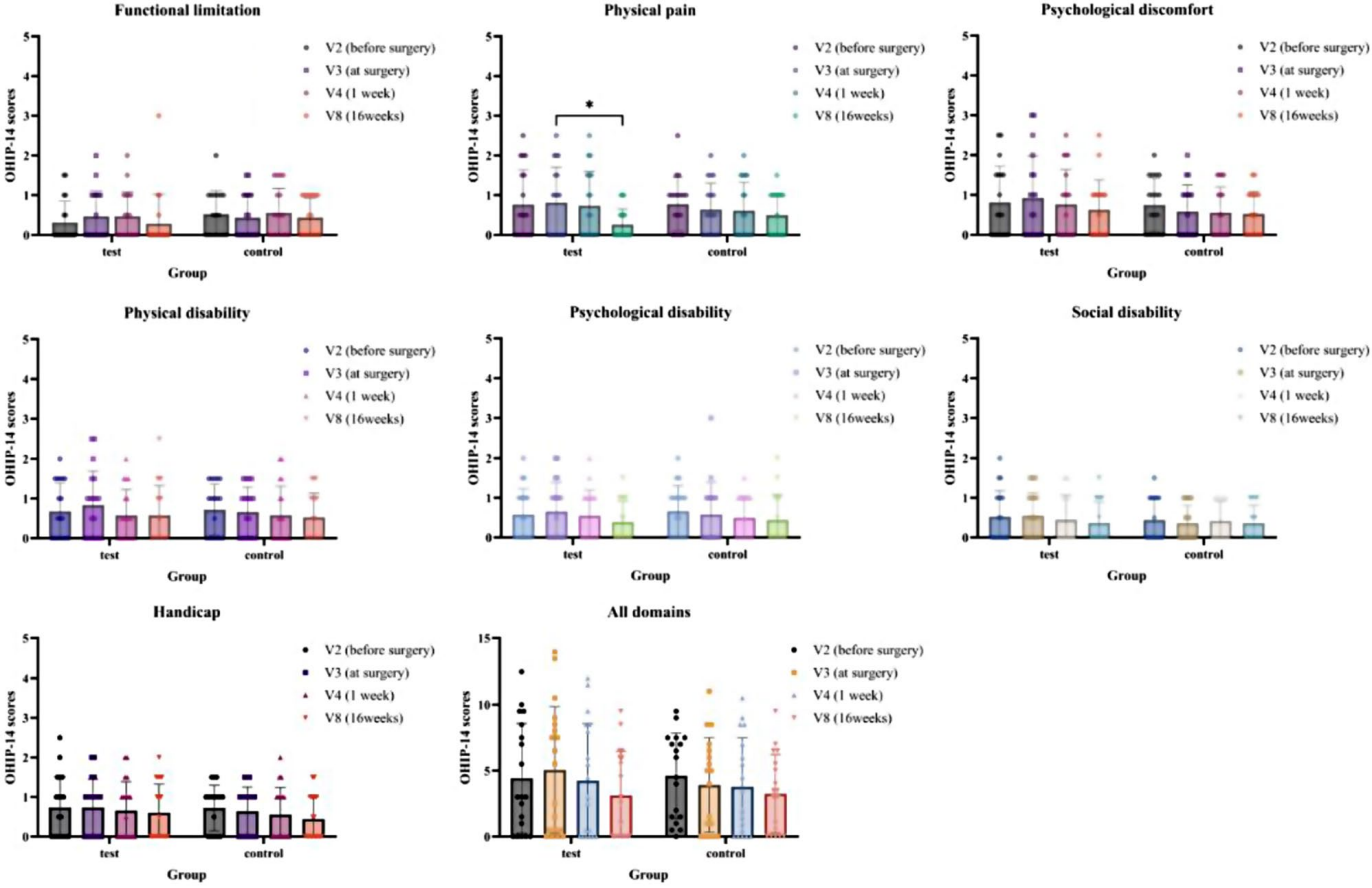
The results of Oral Health Impact Profile-14 (OHIP-14) for seven domains. Data are presented as the mean ± SD. *p < 0.05

In the OHIP-14 results prior to surgery (V2), during surgery (V3), and at 1 (V4) and 16 (V8) weeks post-surgery (as shown in Fig. 10 and Supplementary Table 2), most intragroup comparisons did not reveal any statistically significant differences. The only exception was the physical pain experienced in the test group between V3 (during surgery) and V8 (16 weeks post-surgery), which showed a significant difference (0.82 ± 0.89 vs. 0.26 ± 0.39, *p* < 0.05). However, all intergroup comparisons showed no significant differences.

#### Soft tissue inflammation

**Fig. 11:**
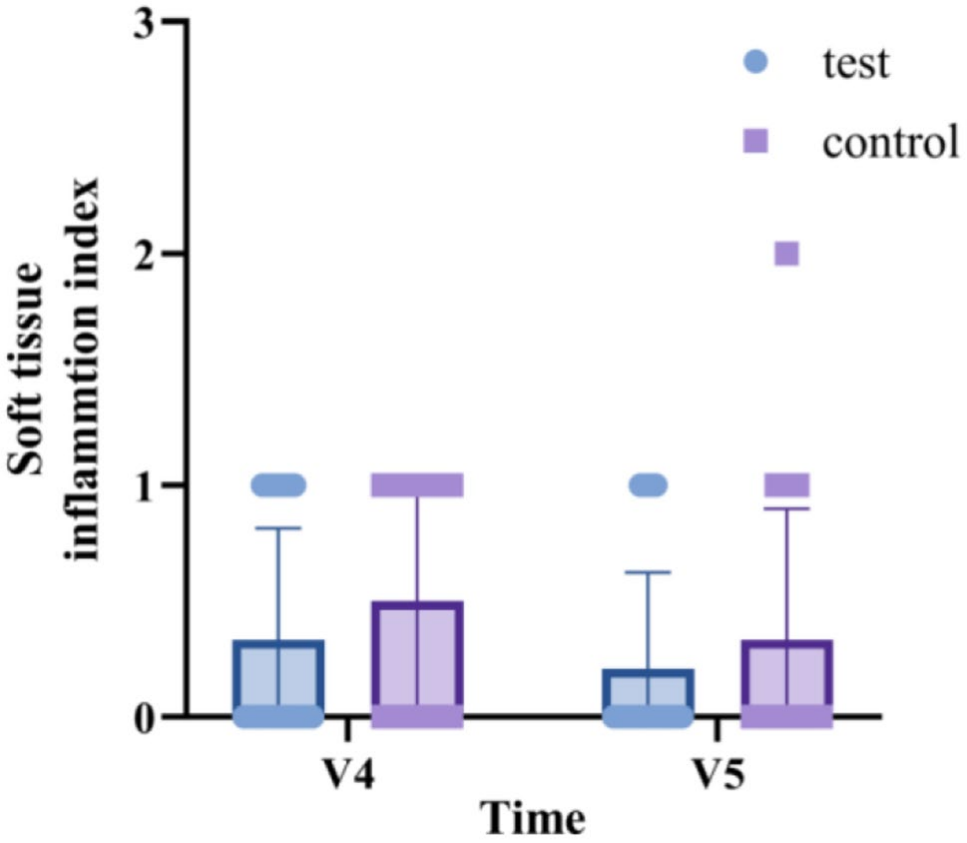
Comparisons of the soft tissue inflammation index between the test and control groups at 1 (V4) and 4 (V5) weeks after implant surgery. Data are presented as the mean ± SD.

The results of the soft tissue inflammation scores are depicted in Fig. 11. No statistically significant differences were observed between the two groups, nor were there any within the individual groups. Throughout the duration of this clinical trial, no soft tissue inflammation scores of ≥ 3, which would indicate a moderate or severe inflammatory response, were recorded.

#### Multivariable linear regression analysis of ISQ value

The results of the multivariable linear regression analysis at both the implant and patient levels are summarized in Table 4. The analysis revealed that age (*p* < 0.05) and diabetes mellitus (DM) (*p* < 0.01) significantly influenced the ISQ value at the time of surgery (V3) at the implant level. On the other hand, age did not significantly affect the ISQ value at the time of surgery at the patient level. However, both DM (*p* < 0.01) and PBA-T (*p* < 0.05) significantly influenced the ISQ value at the time of surgery at the patient level.

**Table 4:** Multivariable linear regression analysis of ISQ value at implant surgery. Continuous variables of site level factors were considered in this analysis

	Implant level		Patient level	
Contributing factors	Regression coefficient	P value	Regression coefficient	P value
Age (< 65 vs. ≥65)	5.894	**0.048**	4.534	0.149
Sex (male vs. female)	-3.381	0.233	-4.657	0.126
Smoking (no vs. yes)	3.388	0.310	4.632	0.185
hypertension	-3.181	0.200	-2.939	0.234
Diabetes mellitus	10.830	**0.003**	9.785	**0.008**
Hyperlipidemia	-2.958	0.331	-1.253	0.707
Hepatitis B virus	4.535	0.552	4.088	0.590
Osteoporosis	5.432	0.336	8.056	0.185
Location	0.500	0.826	0.812	0.721
PPD	-0.494	0.369	-0.800	0.189
REC	-0.693	0.546	0.156	0.907
CAL	NA	NA	NA	NA
Vestibular depth	0.218	0.767	0.377	0.613
PBA-T	0.177	0.066	0.200	**0.042**
PBA-D1/D2	-0.157	0.144	-0.208	0.074
Group	-3.282	0.173	-3.780	0.122
Constant	67.754	< 0.001	67.163	< 0.001

NA: The variable was excluded in the analysis due to multicollinearity with other included variables

PPD, probing pocket depth; REC, recession depth; CAL, clinical attachment level; PBA-T, predicted total bone-to-implant contact area; PBA-D1/D2, predicted D1/D2 bone-to-implant contact area

## Discussion

The aim of this interim study was to investigate the clinical outcomes of HA nano-coated SLA implants in comparison to uncoated SLA implants in the maxillary posterior region. Furthermore, immediate implant placement can significantly reduce the duration of tooth loss and overall treatment time. Consequently, strategies such as surface modification or digital dentistry are necessary to achieve reliable primary and/or secondary stability of implants in areas with poor bone quality or insufficient bone quantity. From this perspective, this clinical trial may suggest a clinical implication for HA nano-coated SLA implants in immediate implant placement at the maxillary posterior region, utilizing a digital workflow of guided surgery.

In our clinical findings, we observed favorable insertion torque and primary stability in both groups. However, ISQ values consistently increased in HA nano-coated SLA implants, while ISQ values slightly decreased during the early osseointegration phase of uncoated SLA implants. This suggests that the increase in hydrophilicity, facilitated by implant surface modification, may enhance osteogenic cell adhesion and osteogenic activity on the implant surface [26, 27]. Several studies have reported that the nanotopography of the HA nano-coated layer improves bone-to-implant contact and promotes better bone formation during osseointegration [28–30]. Furthermore, our previous preclinical study, which focused on the reconstructive treatment of experimental peri-implantitis defects in implants with three different surface characteristics (turned, SLA, and HA nano-coated SLA), revealed that HA nano-coated implants demonstrated a greater bone formation capacity in the reconstructive treatment of peri-implantitis defects compared to turned surface implants [31].

The separation of the HA-coated layer from the implant surface can lead to mechanical or biological issues, such as bacterial infections that occur through gaps caused by the dissolution of the coated layer. Implants with a thick or irregular HA coating are particularly susceptible to these adverse effects [3, 9–11, 32]. These problems can be mitigated by reducing and standardizing the thickness of the HA coating using alternative methods, achieving a micro-nanometer scale. Several previous studies have shown that HA coatings of micro-nano thickness remain firmly attached to the implant surface, preventing detachment. These studies also reported that thin HA coatings can provide sufficient osseointegration of implants, even in the absence of primary stability [33, 34]. Our previous preclinical study [35], using HA-coated implants with a mean thickness of 10 nm through a thermal acid etching technique, also confirmed that the HA nano-coated layers remained histologically intact on the implant surface after placement, and that these HA nano-coated layers improved bone healing during the early osseointegration phase. Furthermore, even if delamination of the coated layer occurs, spontaneous re-osseointegration at the dissolution sites is expected, without any mechanical faults, due to the nanometer scale of the coating. This is anticipated to be significant in the placement of implants at compromised sockets to achieve adequate primary stability.

There is already well-established consensus that immediate implant placement does not prevent ridge volume shrinkage following tooth extraction. These changes are more pronounced on the buccal side compared to the lingual/palatal side [36, 37]. However, these atrophic changes can be effectively mitigated by grafting volume-stable graft materials into the marginal gap between the socket wall and the implant surface. Bone grafting is recommended when the gap exceeds 2 mm [38–41]. In this study, most implants were immediately placed in extraction sockets with severe bone destruction and/or significant soft tissue recession, and the average width of the marginal gaps was over 4 mm. Consequently, reductions in vertical height and ridge volume were observed in both the test and control groups from 4 weeks post-surgery, despite bone grafting with deproteinized bovine bone mineral. Furthermore, most implants needed to be placed toward the buccal or palatal side to achieve sufficient primary stability. These factors appear to have influenced the comparable dimensional changes observed between the buccal and palatal sides.

In this study, the PROMs from subjective questionnaires and the OHIP-14 indicated a higher pain-related response in the test group on the day of surgery. However, this was not significantly greater than the responses of the control group. This finding should be interpreted with caution. The surgical intervention conditions of this study, including patient age, sex, smoking habits, systemic diseases, site level factors, and the number of additional surgeries (bone graft with or without transcrestal sinus augmentation surgery), were not significantly different between the two groups, with the exception of the surface properties of the implants. Previous studies have shown that HA nano-coated implants do not induce postoperative inflammation at surgical sites and exhibit a similar immunological response to pure titanium implants [35, 42]. Our results, using the soft tissue inflammation score as a more objective index, confirmed these findings and observed clinically uneventful healing at nearly all surgical sites. Therefore, the slightly higher pain reported during the placement of HA nano-coated implants at the time of surgery cannot be attributed solely to the implants themselves. Pain is a subjective experience and can be influenced by various factors, including patient characteristics (age, sex, alcohol consumption, smoking, physical and psychological health status) and surgical intervention properties (duration of operation, number and extent of surgical sites, additional surgery) [43–46]. There are several methods for quantifying pain, such as VAS scores, a dichotomous index (yes or no), pain categories (no pain, moderate, severe), and the assessment of painkiller consumption [47]. However, no standardized evaluation methods have been established. Given this complexity, it is challenging to accurately quantify pain. In addition, patients who have previously experienced implant surgery may express their less pain or higher satisfaction with implant surgery compared to those who never experienced, which may act as a factor that makes it difficult to precisely evaluate patient-reported outcomes about HA nano-coated implants. Accordingly, to better understand patient discomfort associated with HA nano-coated implants, further study and comprehensive evaluation, including clinical assessment by the operator, may be necessary.

In the present study, the multivariable linear regression analysis of the ISQ value at the time of implant surgery revealed a correlation between primary stability and factors such as age, DM at the implant level, and DM and PBA-T at the patient level. While there are inconsistent reports regarding the impact of age on implant placement, several studies have reported a high implant survival rate (96.1% over 5 years) in elderly patients [48]. A recent study also found no significant differences in insertion torque and primary stability based on age [49]. Meanwhile, although DM has been regarded as a risk factor that has an interactive relationship with periodontitis [50], favorable clinical outcomes (e.g., high survival and success rates, rigid primary and/or secondary stability) can be expected in patients with well-controlled diabetes, with glycated hemoglobin levels under 8% [51, 52]. These findings do not align with our own, suggesting that the susceptibility of elderly and diabetic patients to advanced periodontal tissue destruction may have influenced these conflicting results. In terms of the effect of PBA-T on the ISQ value at surgery, it was found to be significant at the patient level, but not at the implant level. This discrepancy is likely due to the use of a digital surgical guide for immediate implant placement in this study. The digital surgical guide protocol, which includes evaluating bone quality and quantity, planning virtual implants, and designing surgical guides, allows for optimal primary stability. As a result, it is cautiously suggested that this protocol may have diluted the effect of PBA-T on primary stability at the implant level.

Regarding the long-term outcomes of HA-coated implants, previous studies have reported survival rates exceeding 90% over approximately 10 years. On the other hand, the success rate has been observed to decrease significantly, to around 50%, due to desquamation issues at the interface between the coated layer and the implant surface [53, 54]. However, with advances in HA coating methodology, these earlier complications have been addressed, and the application of thin (micro-nanometer scale) HA-coated implants in clinical practice is currently increasing. Despite several preclinical studies demonstrating the benefits of HA nano-coated implants on osseointegration, clinical trials and long-term observations remain relatively scarce. This study, as an interim outcome of a prospective clinical trial, holds significance in that it confirms the excellent osseointegration potential of HA nano-coated SLA implants. Furthermore, this study is noteworthy as it aims to monitor the survival and success rates of HA nano-coated SLA implants over a 10-year period and evaluate the long-term outcomes in terms of clinical and radiological observations, as well as patient satisfaction. In this study, although adequate primary stability of immediate implant was obtained by using a digital surgical guide, the stability of surgical guide may be affected by the amount of residual bone, uneven extraction socket and guide support methods (tooth or mucosa supported). This may cause inaccuracy of surgical guide stent during ostectomy and fixture placement, and the accuracy of surgical guide stent in immediate implant placement is planned to be evaluated as an additional follow-up study.

The limitation of this study is the heterogeneity in diameter of implants. As a clinical study with 10-year follow up, unlike in healed ridge, optimal diameter of immediate implants had to be selected (4 mm for premolar and 5 mm for molar teeth) for long-term use depending on the conditions of extraction sockets. However, since the implant stability is influenced by not only surface properties but also bone density, implant-bone interface contact, length and diameter of implants [55], it is believed that the interpretation of solely effect of HA nano-coated surface on implant stability should be approached with caution, and further study with unified implant diameter is necessary. Meanwhile, according to the manufacturer’s guideline, an ISQ value of 70 or above indicates high stability [56]. In this study, despite the statistical significance between HA nano-coated and uncoated SLA implants during bone healing, both groups exhibited ISQ value over 70 and considered to be an adequate stability. Therefore, the clinical superiority of HA nano-coated SLA implant to uncoated SLA implant regarding implant stability may be limited. It is considered to be another limitation of this study, and long-term follow up study comparing the clinical prognosis between two groups is needed.

In conclusion, within the limitations of this study, the observations herein indicate that HA nano-coated SLA implants may facilitate superior and more rapid osseointegration in challenging bone conditions. This is particularly evident in cases such as immediate implant placement in the maxillary posterior region, compared to uncoated SLA implants.

### Electronic supplementary material

Below is the link to the electronic supplementary material.

Supplementary Material 1

## Data Availability

The data that support the findings of this study are available from the corresponding authors upon reasonable request.
