# Supplementary material for: A randomized controlled trial of immediate implant placement comparing hydroxyapatite nano-coated and uncoated sandblasted/acid-etched implants using a digital surgical guide
Source: Int J Implant Dent. 2024 Jun 5;10:29. doi: 10.1186/s40729-024-00549-8 (PMC11153479; doi:10.1186/s40729-024-00549-8)
Supplement: Supplementary file 1 — Supplementary Material 1 [file 40729_2024_549_MOESM1_ESM.docx]

Appendices

Supplementary Figure1. Clinical photographs of all test and control groups.


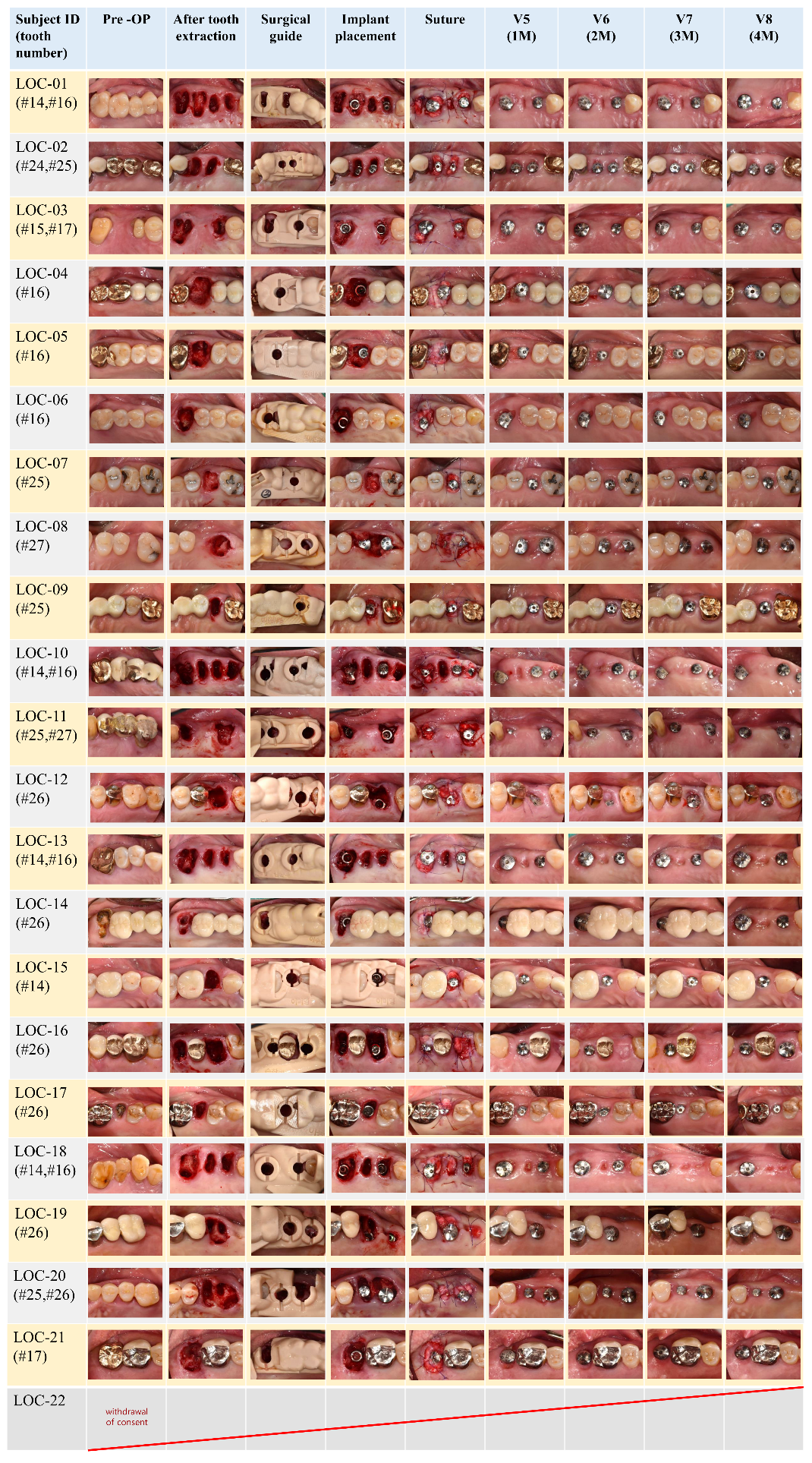


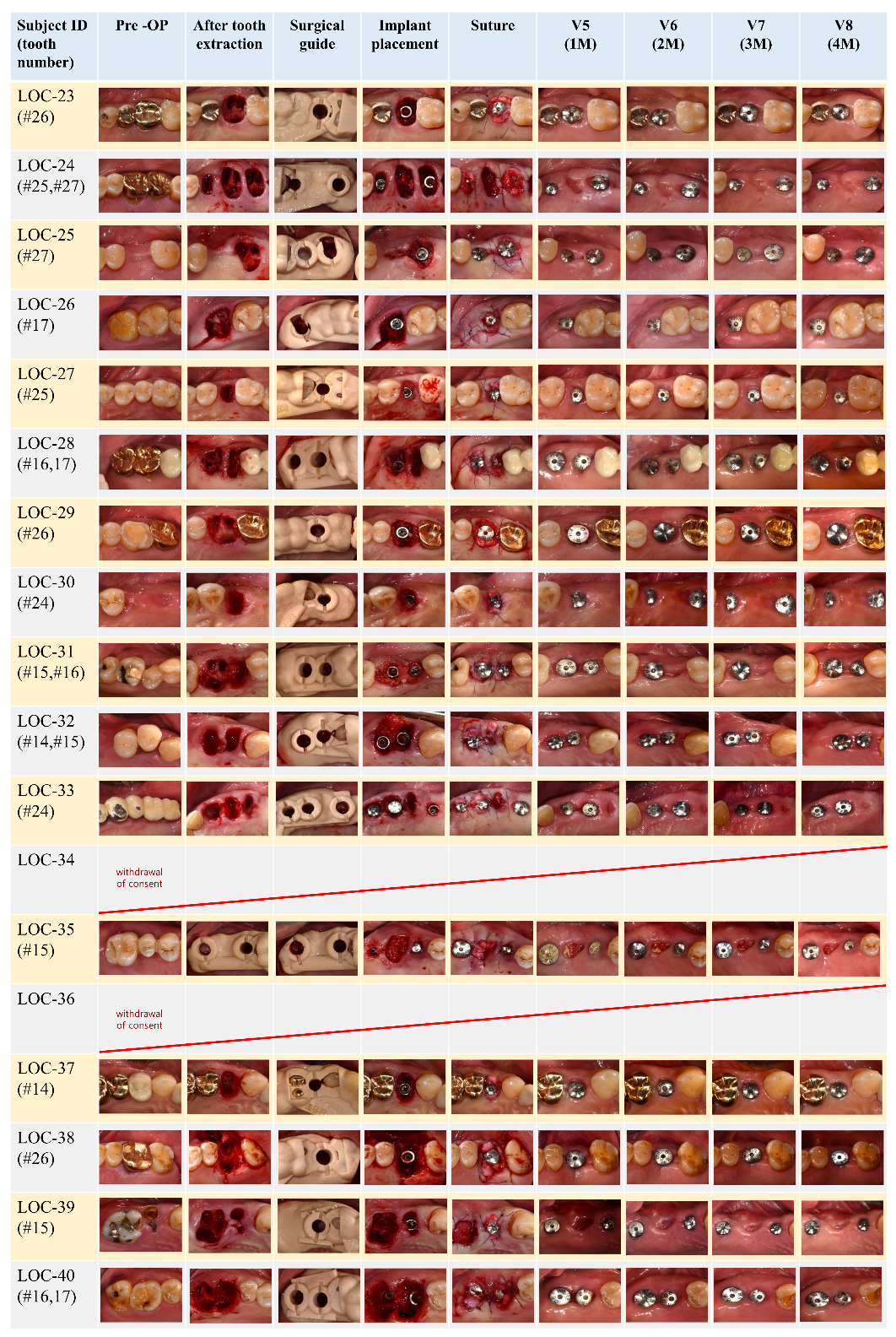


Supplementary Table 1. Concomitant medications other than postoperative antibiotics and analgesics

| ID | Medication | Systemic disease |
| --- | --- | --- |
| LOC-1 | - | None |
| LOC-2 | - | None |
| LOC-3 | Candesartan cilexetil 16mg | Hypertension |
|  | Trimetazidine 2HCl 20mg | cardiovascular disease |
|  | Nicorandil 5mg | angina pectoris |
|  | Avocado-soya unsaponifiabes | arthritis |
| LOC-4 | - | None |
| LOC-5 | Amlodipine camsylate 7.84mg +  Losartan potassium 100mg | Hypertension |
|  | Atorvastatin calcium trihydrate 10.85mg | hyperlipidemia |
| LOC-6 | Calcium carbonate/ 250mg | Osteopenia |
|  | Milk thistle axtract 350mg | liver disease |
|  | Ursodeoxycholic acid 200mg | liver disease |
|  | Azathioprine 50mg | autoimmune disease |
| LOC-7 | - | None |
| LOC-8 | - | None |
| LOC-9 | Metformin hydrochloride 500mg | Diabetes mellitus |
|  | Calcium Carbonate/vitamin D3 250mg | Osteopenia |
|  | Atorvastatin calcium trihydrate 10.85mg | hyperlipidemia |
|  | Sulodexide 250mg | thrombus prevention |
| LOC-10 | Aspirin enteric coated 120.98mg | thrombus prevention |
|  | Rabeprazole sodium 5mg | Excessive secretion of stomach acid |
|  | Ezetimibe 10mg - | hyperlipidemia |
| LOC-11 | - | None |
| LOC-12 | Rosuvastatin calcium 10.4mg | hyperlipidemia |
|  | Metformin hydrochloride 500mg | Diabetes mellitus |
|  | Amlodipine adipate 6.79mg | Hypertension |
| LOC-13 | Metformin hydrochloride 1000mg | Diabetes mellitus |
|  | Evogliptin tartrate 6.869mg | Diabetes mellitus |
|  | Rosuvastatin calcium 5.20mg | hyperlipidemia |
|  | Candesartan cilexetil 16mg | Hypertension |
|  | Acetaminophen 650mg | spinal stenosis, pain reliever drug |
|  | Pregabalin 25mg | spinal stenosis, pain reliever drug |
|  | duloxetine hydrochloride 30mg | depression |
|  | Limaprost 5μg | spinal stenosis, pain reliever drug |
| LOC-14 | - | None |
| LOC-15 | Calcium Carbonate 1250mg | calcium supplement |
|  | Tibolone 2.5mg | menopause |
| LOC-16 | Fenofibrate 160mg | hyperlipidemia |
| LOC-17 | Levothyroxine Sodium hydrate 0.075mg | hypothyroidism |
|  | Rosuvastatin calcium 5.20mg | hyperlipidemia |
| LOC-18 | Levothyroxine Sodium hydrate 0.1mg | hypothyroidism |
|  | atorvastatin calcium trihydrate 10mg - | hyperlipidemia |
| LOC-19 | s-amlodipine besylate 2.5 hydrate 3.74mg | Hypertension |
|  | Rosuvastatin calcium 5mg - | hyperlipidemia |
| LOC-20 | Warfarin sodium 2mg - | valvular disease of the heart |
| LOC-21 | - | None |
| LOC-22 |  |  |
| LOC-23 | - | None |
| LOC-24 | - | None |
| LOC-25 | - | None |
| LOC-26 | aspirin 100mg | cardiovascular disease |
|  | Clopidogrel 75mg | cardiovascular disease |
| LOC-27 | Metformin, Glimepiride | Diabetes mellitus |
| LOC-28 | - | None |
| LOC-29 | - | None |
| LOC-30 | - | None |
| LOC-31 | Losartan potassium 50mg | Hypertension |
|  | Dried Ferrous Sulfate 256mg | anemia |
| LOC-32 | Zemimet SR Tab. 50/500mg | Diabetes mellitus |
|  | Aceclofenac 100mg | shoulder pain |
| LOC-33 | - | None |
| LOC-34 |  |  |
| LOC-35 | Ursodeoxycholic Acid 100mg | Liver disease |
|  | Fimasartan Potassium 60mg | Hypertension |
|  | Godex Cap. | Liver disease |
|  | Pitavastatin Calcium 2mg | hyperlipidemia |
|  | Clopidogrel 75mg | thrombus prevention |
|  | Hydrochlorothiazide 25mg | Hypertension |
| LOC-36 |  |  |
| LOC-37 | - | None |
| LOC-38 | Olmesartan Medoxomil 20mg | Hypertension |
| LOC-39 | - | None |
| LOC-40 | - | None |

“-“ indicates no medication for any systemic disease.

Supplementary Table 2. The value for the seven domains of OHIP-14.

|  |  | test | | | | control | | | |
| --- | --- | --- | --- | --- | --- | --- | --- | --- | --- |
| Domain of OHIP-14 |  | V2 | V3 | V4 | V8 | V2 | V3 | V4 | V8 |
| Functional limitation | Mean (SD) | 0.32 (0.53) | 0.47 (0.63) | 0.47 (0.61) | 0.29 (0.73) | 0.53 (0.58) | 0.44 (0.57) | 0.56 (0.62) | 0.44 (0.48) |
|  | Median (IQR) | 0 (0 -0.5) | 0 (0-1) | 0 (0-1) | 0 (0-0) | 0.5 (0-1) | 0 (0-1) | 0.5 (0-1.125) | 0.25 (0-1) |
| Physical pain | Mean (SD) | 076 (0.87) | 0.82 (0.89) | 0.74 (0.86) | 0.26 (0.39) | 0.78 (0.67) | 0.64 (0.66) | 0.61 (0.72) | 0.50 (0.54) |
|  | Median (IQR) | 0.5 (0.0-1.5) | 1 (0-1.5) | 0.5 (0-1.125) | 0.25 (0-1.5) | 1 (0-1) | 0.5 (0-1.125) | 0.25 (0-1.5) | 0.25 (0-1) |
| Psychological discomfort | Mean (SD) | 0.82 (0.90) | 0.92 (1.06) | 0.76 (0.87) | 0.63 (0.74) | 0.75 (0.67) | 0.58 (0.67) | 0.56 (0.64) | 0.53 (0.56) |
|  | Median (IQR) | 0.5 (0-1.5) | 0.5 (0-1.5) | 0.5 (0-1.5) | 0.5 (0-1) | 0.75 (0-1.5) | 0.25 (0-1) | 0 (0.25-1.125) | 0.5 (0-1) |
| Physical disability | Mean (SD) | 0.68 (0.71) | 0.84 (0.85) | 0.58 (0.65) | 0.58 (0.75) | 0.72 (0.65) | 0.67(0.62) | 0.58 (0.73) | 0.53 (0.61) |
|  | Median (IQR) | 0.5 (0-1.5) | 1 (0-1.5) | 0.5 (0-1) | 0 (0-1) | 1 (0-1.5) | 0.75 (0-1.125) | 0.25 (0-1.125) | 0.25 (0-1) |
| Psychological disability | Mean (SD) | 0.58 (0.65) | 0.66 (0.73) | 0.55 (0.64) | 0.40 (0.52) | 0.67 (0.64) | 0.58 (0.81) | 0.50 (0.54) | 0.44 (0.64) |
|  | Median (IQR) | 0.5 (0-1) | 0.5 (0-1) | 0 (0-1) | 0 (0-1) | 0.75 (0-1) | 0 (0-1) | 0.25 (0-1) | 0 (0-1) |
| Social disability | Mean (SD) | 0.53 (0.66) | 0.55 (0.58) | 0.45 (0.62) | 0.37 (0.52) | 0.44 (0.54) | 0.36 (0.45) | 0.42 (0.49) | 0.36 (0.45) |
|  | Median (IQR) | 0 (0-1) | 0.5 (0-1) | 0 (0-1) | 0 (0-1) | 0 (0-1) | 0 (0-1) | 0 (0-1) | 0 (0-1) |
| Handicap | Mean (SD) | 0.74 (0.79) | 0.74 (0.71) | 0.66 (0.73) | 0.61 (0.72) | 0.72 (0.58) | 0.64 (0.61) | 0.56 (0.68) | 0.44 (0.57) |
|  | Median (IQR) | 0.5 (0-1.5) | 1 (0-1) | 0.5 (0-1) | 0 (0-1.5) | 1 (0-1) | 1 (0-1) | 0 (0-1) | 0 (0-1) |
| Overall domains | Mean (SD) | 4.42 (4.15) | 5.05 (4.80) | 4.24 (4.32) | 3.13 (3.33) | 4.61 (3.25) | 3.92 (3.57) | 3.78 (3.71) | 3.25 (2.96) |
|  | Median (IQR) | 3 (0-8.5) | 5.5 (0.5-8.5) | 3 (0-8.5) | 2.5 (0-6) | 5.25 (1.375-7.5) | 4.5 (0-6.625) | 2.75 (0-7.375) | 3.25 (0-5.75) |
